# Ginkgolide A improves the pleiotropic function and reinforces the neuroprotective effects by mesenchymal stem cell-derived exosomes in 6-OHDA-induced cell model of Parkinson’s disease

**DOI:** 10.18632/aging.204526

**Published:** 2023-02-20

**Authors:** William Shao-Tsu Chen, Tzu-Ying Lin, Chia-Hua Kuo, Dennis Jine-Yuan Hsieh, Wei-Wen Kuo, Shih-Chieh Liao, Hui-Chuan Kao, Da-Tong Ju, Yu-Jung Lin, Chih-Yang Huang

**Affiliations:** 1 Department of Psychiatry, Tzu Chi General Hospital, Hualien 97004, Taiwan; 2 School of Medicine Tzu Chi University, Hualien 97004, Taiwan; 3 Cardiovascular and Mitochondrial Related Disease Research Center, Hualien Tzu Chi Hospital, Buddhist Tzu Chi Medical Foundation, Hualien 970, Taiwan; 4 Laboratory of Exercise Biochemistry, University of Taipei, Taipei, Taiwan; 5 Clinical Laboratory, Chung Shan Medical University Hospital, Taichung 402, Taiwan; 6 School of Medical Laboratory and Biotechnology, Chung Shan Medical University, Taichung, Taiwan; 7 Department of Biological Science and Technology, China Medical University, Taichung, Taiwan; 8 Graduate Institute of Chinese Medical Science, China Medical University, Taichung, Taiwan; 9 Department of Public Health, Tzu Chi University, Buddhist Tzu Chi Medical Foundation, Hualien 970, Taiwan; 10 Department of Neurological Surgery, Tri-Service General Hospital, National Defense Medical Center, Taipei, Taiwan; 11 Graduate Institute of Biomedical Sciences, China Medical University, Taichung 404, Taiwan; 12 Department of Biological Science and Technology, Asia University, Taichung, Taiwan; 13 Center of General Education, Buddhist Tzu Chi Medical Foundation, Tzu Chi University of Science and Technology, Hualien 970, Taiwan; 14 Department of Medical Research, China Medical University Hospital, China Medical University, Taichung 404, Taiwan

**Keywords:** Ginkgolide A, Wharton’s jelly, mesenchymal stem cell, 6-hydroxydopamine, parkinson’s disease, exosomes

## Abstract

Parkinson’s disease (PD) is a common disorder attributed to the loss of midbrain dopamine (mDA) neurons and reduced dopamine secretion. Currently, the treatment regimes for PD comprise deep brain stimulations, however, it attenuates the PD progression marginally and does not improve neuronal cell death. We investigated the function of Ginkgolide A (GA) to reinforce Wharton’s Jelly-derived mesenchymal stem cells (WJMSCs) for treating the *in vitro* model of PD. GA enhanced the self-renewal, proliferation, and cell homing function of WJMSCs as assessed by MTT and transwell co-culture assay with a neuroblastoma cell line. GA pre-treated WJMSCs can restore 6-hydroxydopamine (6-OHDA)-induced cell death in a co-culture assay. Furthermore, exosomes isolated from GA pre-treated WJMSCs significantly rescued 6-OHDA-induced cell death as determined by MTT assay, flow cytometry, and TUNEL assay. Western blotting showed that apoptosis-related proteins were decreased following GA-WJMSCs exosomal treatment which further improved mitochondrial dysfunction. We further demonstrated that exosomes isolated from GA-WJMSCs could restore autophagy using immunofluorescence staining and immunoblotting assay. Finally, we used the alpha-synuclein recombinant protein and found that exosomes derived from GA-WJMSCs led to the reduced aggregation of alpha-synuclein compared to that in control. Our results suggested that GA could be a potential candidate for strengthening stem cell and exosome therapy for PD.

## INTRODUCTION

Parkinson’s disease (PD) was first characterized two hundred years ago and is one of the most common neurodegenerative disorders. Until now, it was estimated that more than 2% of the older population suffers from Parkinson’s disease [1]. This percentage is expected to rise with the aging of the population worldwide and a few reports state that PD would become a pandemic [2]. The pathological features of PD include the loss of midbrain dopamine (mDA) neurons or neuronal cell loss in the substantia nigra (SN), decreased dopamine secretion, and Lewy body accumulation in other brain tissues, thereby exacerbating motor functions [3–7]. The pathophysiology of PD remains unknown but may involve genetic and environmental risks. The most effective treatments for PD involve deep brain stimulations through surgery or taking some enzyme inhibitors and levodopa [8]. However, long-term drug usage may reduce its efficacy and lead to side effects involving involuntary motor action and dyskinesia, which affects patients’ quality of life.

Studies on stem cell therapy in PD have been popular for the past decades. Mesenchymal stem cells (MSCs) are the most appropriate for stem cell therapy because of two main characteristics: the immuno-modulatory function and the differentiation ability [9]. MSCs can be isolated from bone marrow, adipose tissues, umbilical cord Wharton’s Jelly (WJ), and even the dental pulp, which are classified as “mesenchymal resources” and have inspired researchers to find their abilities in cell transplantation application for PD. The secretome of stem cells has been found to play an important role in several degenerative diseases [10]. Increasing evidence has revealed that extracellular vesicles (EVs) play multiple roles in various physiological conditions, especially through cell–cell communication. EVs from MSCs include microvesicles, apoptotic bodies, and exosomes. The exosomes consist of genetic substances (DNA, RNA, and non-coding RNA), proteins, enzymes, and some growth factors [11]. Exosomes from MSCs can regulate different cell signaling pathways in target cells and promote their biological activities by transmitting information and content to improve disease conditions [12].

*Ginkgo biloba* is a common herbal drug widely used in China, Europe, and other regions. The extract of *Ginkgo biloba* contains ginkgolides, which comprise Ginkgolide A (GA), Ginkgolide B (GB), Ginkgolide C (GC), Ginkgolide J (GJ), Ginkgolide K (GK), and other ginkgolides [13]. GA has been shown to inhibit α-amino-3-hydroxy-5-methyl-4-isoxazolepropionic acid receptors and N-methyl-D-aspartate receptor-induced neuronal depolarization [14]. Furthermore, it was suggested to have a role in anti-oxidant, anti-inflammatory mechanisms, and alleviating the symptoms of Alzheimer’s disease [15–17]. Nevertheless, the function of GA in MSCs remains to be elucidated.

Here, we aimed to understand the role of GA in Wharton’s Jelly MSCs to aid in restoring the 6-hydroxydopamine (6-OHDA)-induced neuronal cell death. Hence, we assessed the self-renewal, homing, and exosome-mediated functions of MSCs treated with GA, which may be a potential compound to be used in stem cell or secretome therapy.

## MATERIALS AND METHODS

### Cell culture

Wharton’s Jelly MSCs were obtained from Cellular Engineering Technologies Inc. (CET, Coralville, IA, USA) and SH-SY5Y cells obtained from the American Type Culture Collection (Manassas, VA, USA) were cultured in Dulbecco’s modified essential media (Life Technologies) with 10% fetal bovine serum (FBS, Hyclone Thermo Fisher Scientific) and 1% penicillin-streptomycin (Gibco). Cells were incubated at 37°C with 5% CO_2_ in a humidified incubator.

### MTT (3-(4,5-Dimethylthiazol-2-yl)-2,5-diphenyltetrazolium bromide) assay

Cells were seeded in 24-well plates (1–3 × 10^4^ cells/well) and cultured in a complete DMEM medium for 12 h. Further, they were treated with 6-OHDA for 6–48 h at 37°C. Cells were washed twice with phosphate-buffered saline (PBS) and incubated with the MTT reagent (Invitrogen^™^) for 2 h. At the end of the treatment, cells were incubated in Dimethyl sulfoxide and shaken for 10–15 min. Cell viability was analyzed as the optical density (OD) was determined spectrophotometrically at a wavelength of 570 nm.

### Transwell co-culture assay

To assess the effect of WJMSCs on neuroblast cells, *in vitro* co-culture assay was performed where WJMSCs (2 × 10^4^ cells) were placed in the upper chamber (0.4 μm), and SH-SY5Y (3 × 10^4^ cells) cells were seeded in the lower well (24 well plate). SH-SY5Y cells were treated with 6-OHDA and incubated at 37°C for 24–48 h. The SH-SY5Y cell viability were to be determined.

### Transwell homing assay

To examine the *in vitro* homing ability, WJMSCs (1 × 10^4^ cells) were seeded in the upper chamber of transwell migration chambers and SH-SY5Y (3 × 10^4^) cells in the lower well containing 500 μL of medium with 6-OHDA and incubated at 37°C for 24 h. Later, cells were removed by a cotton swab from the transwell membrane and cells on the surface of the well were fixed using 4% paraformaldehyde (PFA). The fixed cells were washed in PBS and stained with crystal violet. Cells were counted and the migration ability was expressed as fold-change compared to control.

### Flow cytometry assay for marker expression and apoptosis/cell cycle assessment

SH-SY5Y or WJMSCs were washed twice with PBS then incubated with 5 μL FITC Annexin V and propidium iodide reagent for 15 min at RT in the dark according to the instruction. The apoptotic cells were analyzed using flow cytometry where 10,000 events were acquired in BD FACSLyric^™^ system. For marker expression, WJMSCs with or without GA treatment were collected and fixed with 4% paraformaldehyde solution then washed twice with PBS. WJMSCs were then stained with Vimentin (Santa Cruz; sc-32322, 1:100) for 30 min and incubated with secondary FITC-conjugated goat anti-mouse IgG (Invitrogen; A11001, 1:100) in the dark for 30 min. The cells were acquired in BD FACSLyric^™^ system.

### Exosome isolation assay

Exosomes were collected from the culture medium of WJMSCs using an Exosome Purification Kit (Exo-spin^™^) as per the manufacturer’s protocol. The culture medium was centrifuged at 300 × *g* for 10 min to remove cells and debris. The supernatant was then transferred to a fresh centrifuge tube and spun at 20,000 × *g* for 30 min to remove any cell debris. The exosomes were then precipitated at 4°C for 24 h. The exosome-containing pellet (resuspended in 200 μL PBS) was added to the column and centrifuged at 50 × *g* for 60 s.

### Identification and characterization of exosomes and immunoblotting assay

Cells were lysed using RIPA buffer containing 1% phenylmethylsulfonyl fluoride (PMSF), 20 mM Tris-HCl (pH 7.5), 150 mM NaCl, 1 mM Na_2_ EDTA, 1% NP-40, and 1% sodium deoxycholate. The protein concentrations of the cell lysate or exosomes were estimated using the Bradford Reagent (BioRad #500). For the western blotting assay, 50 μg of the total lysates were separated using SDS PAGE, and proteins were transferred onto membranes. The membranes were incubated with primary antibodies at 4°C for 16 h: anti-pAKT (Santa Cruz; sc-514032, 1:1000), anti-AKT (Santa Cruz; sc-5298, 1:1000), anti-PCNA (Santa Cruz; sc-56, 1:1000), anti-CyclinE (Santa Cruz; sc-25303, 1:1000), anti-CyclinB (Santa Cruz; sc-752, 1:1000), anti-CyclinD (Santa Cruz; sc-246, 1:1000), anti-GAPDH (Santa Cruz; sc-32233, 1:1000), anti-OCT4 (Cell signaling; #2750, 1:1000), anti-Nanog (Cell signaling; #4903, 1:1000), anti-CXCR4 (Millipore; AB1847, 1:1000), anti-CXCR7 (Abcam; ab72100, 1:1000), anti-TSG101 (Abcam; ab83, 1:1000), anti-CD81 (Abcam; ab79559, 1:1000), anti-Caspase-9 (Santa Cruz; sc-8355, 1:1000), anti-Caspase-3 (Santa Cruz; sc-7148, 1:1000), anti-Bcl-2 (BD; 610539, 1:1000), anti-Cytochrome C (Santa Cruz; sc-13560, 1:1000), anti-BAX (Santa Cruz; sc-7480, 1:1000), Total OXPHOS Rodent WB Antibody Cocktail (Abcam; ab110413, 1:1000), anti- Beclin-1 (Cell Signaling; #3738, 1:1000), anti-β-actin (Santa Cruz; sc-47778, 1:1000), anti-p62 (Cell Signaling; #5114, 1:1000), anti-LC3B (Cell Signaling; #2775, 1:1000). HRP-conjugated secondary antibodies (Santa Cruz; sc-2357 and sc-358917, 1:5000-10,000) were added to the membranes and then detected using the ECL reagents in a UVP imaging system (UVP ChemStudio) as previously described [18].

### Immunofluorescence staining

Briefly, alpha-synuclein (5 μM, active, Pre-Formed Fibrils, GeneTex) were treated to the SH-SY5Y cell along or not along with 6-OHDA for 24 hours. The cells were then fixed in 4% PFA, washed twice with PBS, and immunostained with a specific primary antibody (1:50). The cells were then incubated with a fluorescence conjugated secondary antibody (1:50) then used the DAPI mounting gel (Abcam, ab104139) to cover the slides and visualized with a fluorescence microscope (BX40, Olympus) as previously described [19].

### Statistical analyses

All experiments were repeated at least three times and statistical analysis was evaluated using SigmaPlot 12.5 software (Systat Software, Inc., San Jose, CA, USA). Student *t*-test or one-way analysis of variance (ANOVA) was used to analyze the differences in the data. *P* < 0.05 was considered as significant.

### Data availability statement

The data generated from this manuscript are available from the corresponding author on reasonable request.

## RESULTS

### GA promotes proliferation and stemness of WJMSCs

**Figure 1:**
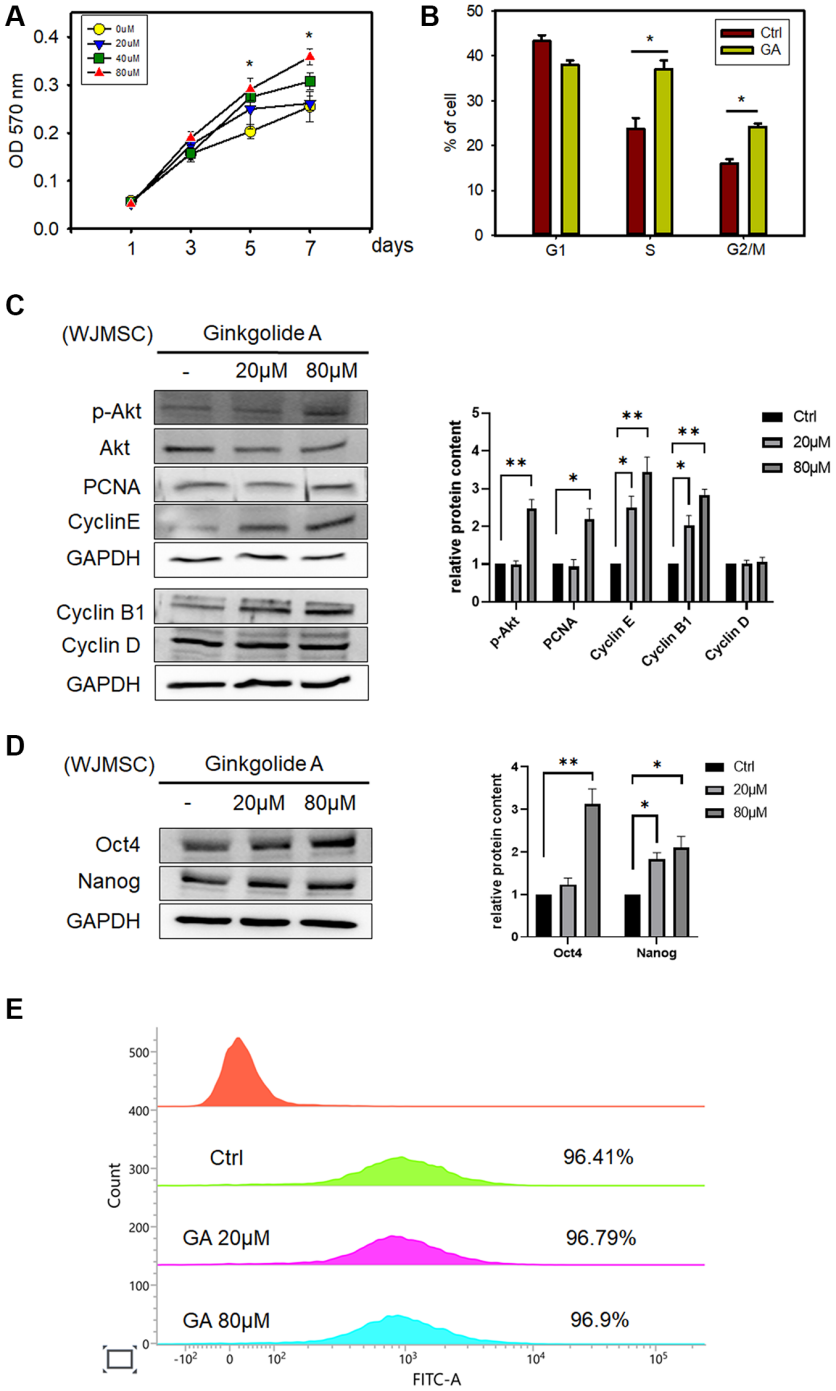
**Ginkgolide A (GA) promotes WJMSC proliferation and stemness.** (**A**) SH-SY5Y cell viability was determined by MTT assay after exposure to 20, 40, and 80 μM GA for 1, 3, 5, and 7 days. The data represent the mean ± standard deviation. ^*^P < 0.05 vs. 0 μM GA. (**B**) Cell cycle assessed by flow cytometry after exposure to 80 μM GA. (**C**) Proliferation and cell cycle-related proteins p-AKT, PCNA, Cyclin E, Cyclin B1, and Cyclin D were examined after GA treatment in WJMSCs by western blot analysis. (**D**) Western blotting of stemness-related markers OCT4 and Nanog after GA treatment in WJMSCs. (**E**) Vimentin expression of WJMSC after exposure to 20 μM, 80 μM GA. ^*^P < 0.05, ^**^P < 0.01 vs. ctrl.

The potential effect of GA on WJMSC proliferation was investigated by treating with different concentrations of GA. After 5 d of treatment, GA (80 μM) promoted WJMSCs proliferation (Figure 1A) as examined by MTT assay. We further assessed the changes in cell cycle phases after GA treatment and found that it could change the cell cycle distribution in S and G2/M phases as compared to that in controls (Figure 1B). In addition, the expression of proliferation markers, p-AKT, PCNA, Cyclin E, and Cyclin B1, was increased after GA treatment (Figure 1C). GA also promoted the stemness markers, OCT4 and Nanog, in WJMSCs (Figure 1D) and maintained the MSC marker Vimentin expression (Figure 1E). These findings indicated that GA acts in improving the self-renewal ability of the stem cells.

### GA enhances the homing ability of WJMSCs

**Figure 2:**
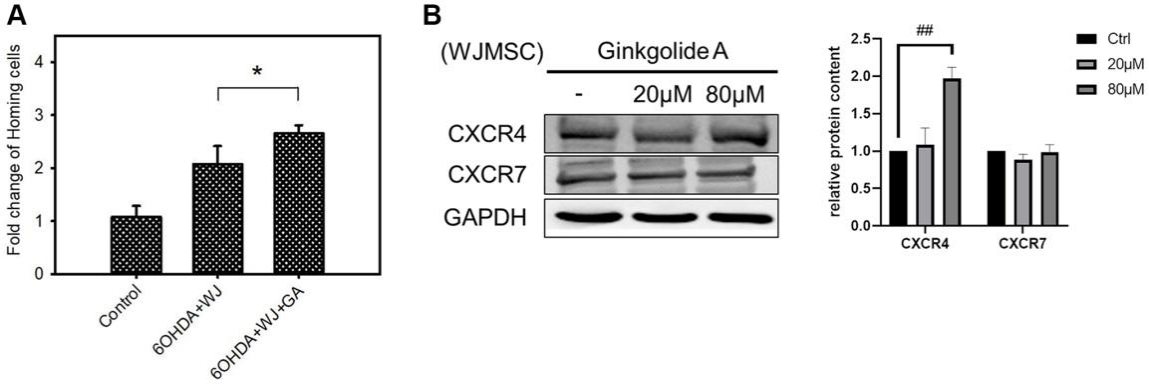
**Effect of Ginkgolide A (GA) on WJMSCs homing capacity.** (**A**) Transwell co-culture migration assay performed to examine the homing function. (**B**) CXCR4 and CXCR7 expression were determined after 20, 80 μM GA treatment in WJMSCs. The data are expressed as cell survival (fold-change) and are presented as the mean ± standard deviation. ^*^P < 0.05 vs. control (without 6-hydroxydopamine (6-OHDA) damage), ^##^*P* < 0.01 vs. ctrl.

In order to explore the multiple functions of GA in stem cells, we further examined the homing ability in GA pre-treated WJMSC (24 h) by co-culturing with SH-SY5Y cells in a 6-OHDA damage model. WJMSCs pre-treated with 80 μM GA was seeded into the inserts (2 × 10^5^ cells/insert) of a transwell. SH-SY5Y cells were pre-plated into the lower wells with 100 μM of 6-OHDA. The migrating WJMSCs counted after 24 h revealed that GA pre-treated WJMSCs displayed higher migrating capacity compared to that of the untreated controls (Figure 2A). Moreover, GA enhanced CXCR4 expression instead of CXCR7 in WJMSCs (Figure 2B). These results suggested that GA could promote WJMSCs homing ability through the SDF-1/CXCR4 axis which is in the line with previous studies that showed preconditioned MSCs required CXCR4 for cell migration whereas CXCR7 for survival in damage area [20, 21].

### GA pre-treated WJMSCs effectively protect SH-SY5Y cells from 6-OHDA induced apoptosis

**Figure 3:**
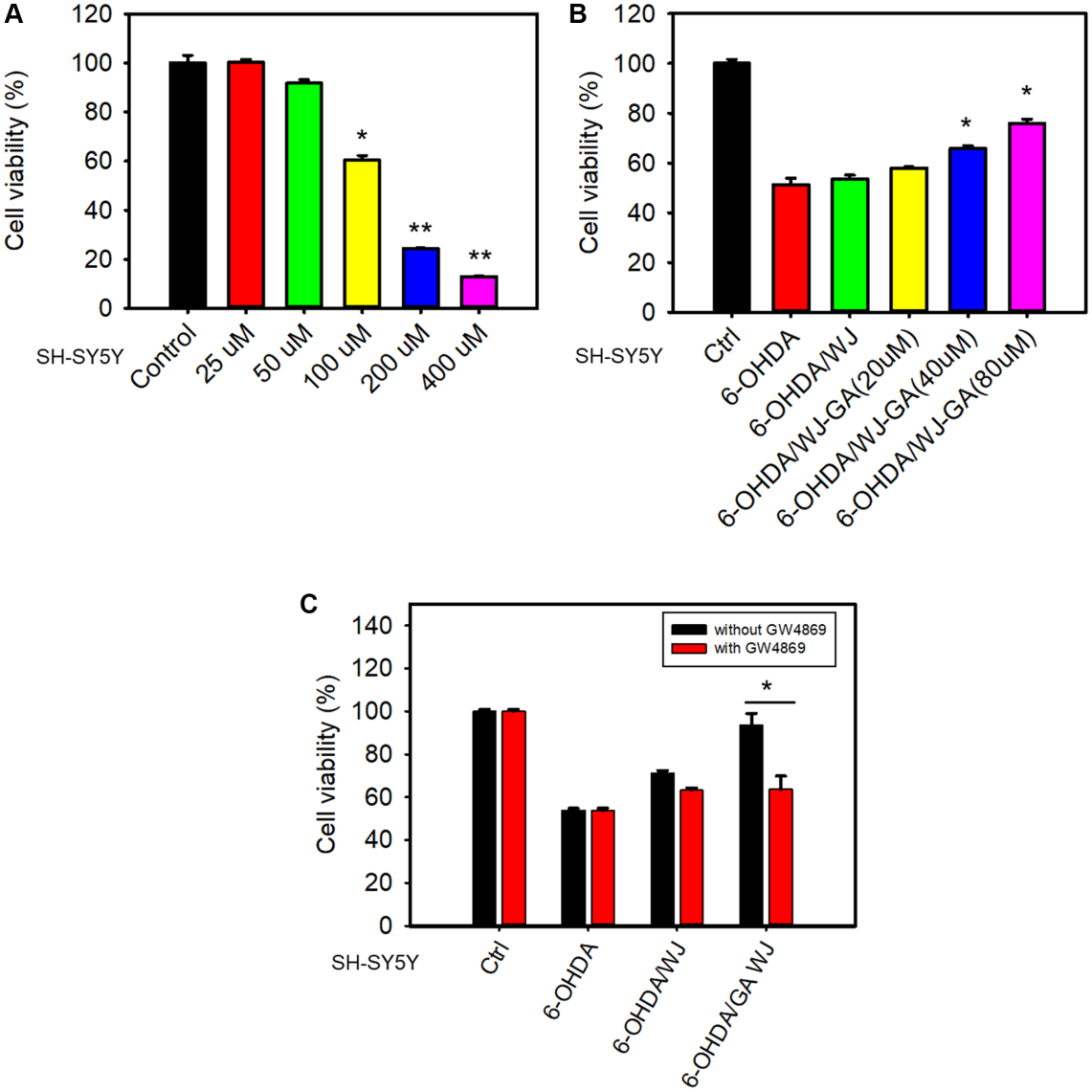
**GA reinforces WJMSCs to improve SH-SY5Y cell viability in a 6-OHDA damage model.** (**A**) 6-OHDA-induced cell death was determined using an MTT assay. (**B**) Transwell co-culture assay was employed to determine the rescue ability of GA-promoted WJMSCs in the 6-OHDA damage model. (**C**) The effects of exosome inhibitor GW4869 on GA-reinforced WJMSCs protective ability were performed. The data is represented as the mean values ± standard deviation. Significance ascribed as ^*^P < 0.05, ^**^P < 0.01 vs. control.

6-OHDA is a well-known chemical that induces the death of dopaminergic neurons by oxidative stress. To determine whether GA can promote the WJMSCs to rescue SH-SY5Y cells in the 6-OHDA damage model, we performed transwell co-culture assay as previously described [22]. 6-OHDA decreased SH-SY5Y cell viability and the concentration of 100 μM was chosen for further studies (Figure 3A). Further, GA pre-treatment in WJMSCs significantly mitigated the death of SH-SY5Y cells under 6-OHDA stimulation (Figure 3B). These findings suggested that GA pre-treated WJMSCs might repair the damage through secretomes, cytokines, or exosomes. To further clarify the mode of action, we utilized GW4869, an inhibitor of exosome generation which blocked the exosome production, in the co-culture assay. Here, a reduction in the GA pre-treated WJMSCs-dependent protection was observed after GW4869 treatment (Figure 3C). These data suggest that exosomes play an important role in GA-mediated WJMSCs therapeutic function.

### Effects of GA pre-treated WJMSC-derived exosomes on the 6-OHDA damage model

**Figure 4:**
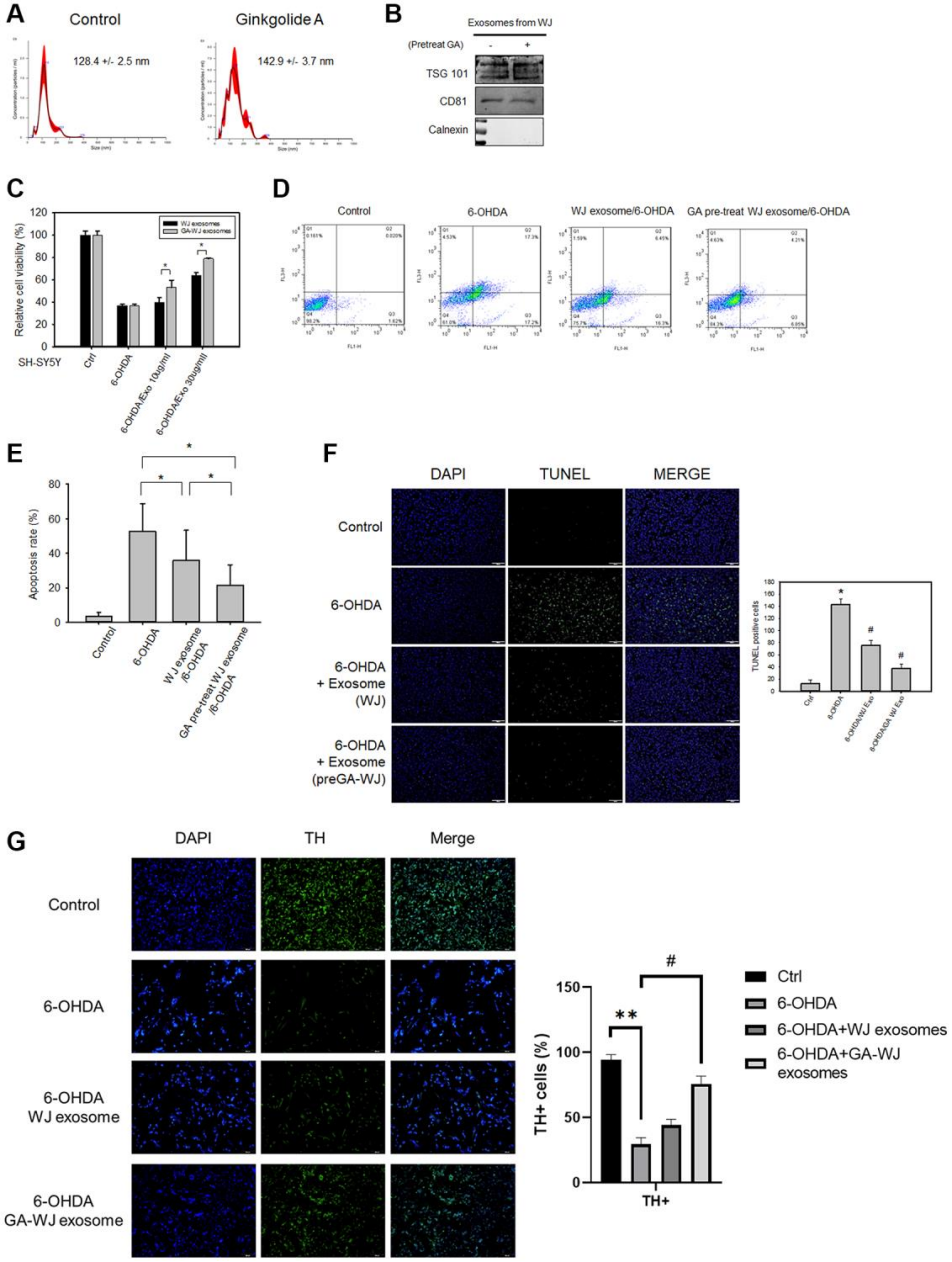
**The therapeutic functions and characteristics of exosomes isolated from GA pre-treated WJMSCs.** (**A**) Size distributions of exosomes as measured by NanoSight NS300 with mean values. (**B**) Exosome positive/negative markers TSG101, CD81, and Calnexin were measured by western blotting assay. (**C**) MTT assay was used to examine the SH-SY5Y cell viability. (**D**) Representative images of flow cytometry showed the cell apoptosis stained with Annexin V-FITC/PI apoptosis detection kit. (**E**) Statistical analysis of the apoptotic data from (**D**). (**F**) TUNEL assay was used to stain the DNA breaks to examine the therapeutic function of exosomes isolated from GA pre-treated WJMSCs. Data are presented as mean ± standard deviation. (**G**) TH staining after exosome treatment isolated from GA pre-treated WJMSCs under 6-OHDA damage. ^*^P < 0.05 vs. control and ^#^*P <* 0.05 vs. 6-OHDA groups.

To gain insight into the WJMSC exosome function enhanced by GA, exosomes were isolated from WJMSCs with or without 80 μM GA pre-treatment, and their diameters were examined by the NanoSight (NS300, Malvern Panalytical‎) (Figure 4A). Further, the presence of the exosome markers tumor susceptibility gene 101 (TSG101) and CD81 and the negative marker calnexin was confirmed (Figure 4B). We found that the exosomes derived from GA pre-treated WJMSCs enhanced SH-SY5Y cell viability under 6-OHDA treatment than that in control WJMSCs using MTT assay (Figure 4C). Flow cytometry and TUNEL assay also demonstrated that exosomes derived from GA pre-treated WJMSCs markedly reduced SH-SY5Y cell apoptosis ratio compared to that of control (Figure 4D–4F). Tyrosine hydroxylase (TH) also revealed that TH-positive cells were decreased under 6-OHDA treatment but could be preserved by exosomes derived from GA pre-treated WJMSCs (Figure 4G).

### GA pre-treated WJMSC-derived exosomes suppress apoptosis markers and impair mitochondrial homeostasis

**Figure 5:**
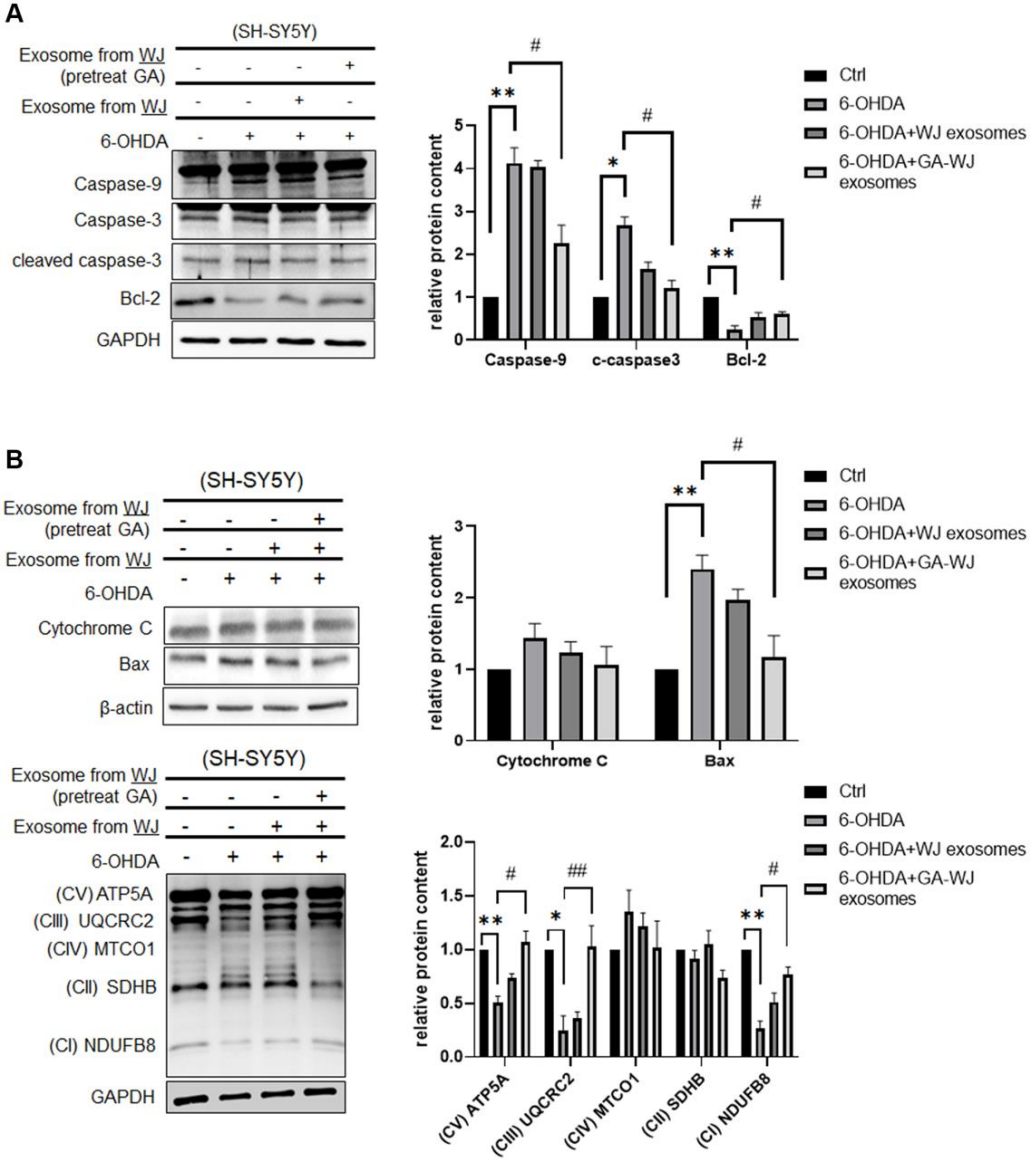
**Expression of apoptosis-related and OXPHOS proteins in SH-SY5Y cells after GA pre-treated WJMSCs derived exosome treatment.** (**A**) Apoptosis-related and anti-apoptotic proteins Caspase-9, Caspase-3, and Bcl-2 were measured by western blotting assay. (**B**) Expression of Cytochrome C, Bax, and OXPHOS protein (ATP5A, UQCRC2, MTCO1, SDHB, NDUFB8) were determined by western blotting. ^*^P < 0.05, ^**^P < 0.01 vs. control and ^#^*P <* 0.05, ^##^*P* < 0.01 vs. 6-OHDA groups.

The above results illustrated that GA displays multiple functions and repairs the 6-OHDA damage cell model. We further investigated the mechanism to support the functional assays. Exosomes isolated from GA pre-treated WJMSCs could decrease apoptosis-related protein Caspase-9 and cleaved Caspase-3 expression but increased anti-apoptosis protein Bcl-2 expression (Figure 5A). Moreover, it also showed that exosomes isolated from GA pre-treated WJMSCs can reduce cytochrome C and Bax expression under 6-OHDA stimulation (Figure 5B). Therefore, we examined the OXPHOS markers and identified that exosomes isolated from GA pre-treated WJMSCs can restore the mitochondrial homeostasis observed during the 6-OHDA damage in SH-SY5Y cells (Figure 5B, lower panel).

### GA pre-treated WJMSC-derived exosomes recover excessive autophagy in the 6-OHDA model and promote alpha-synuclein clearance

**Figure 6:**
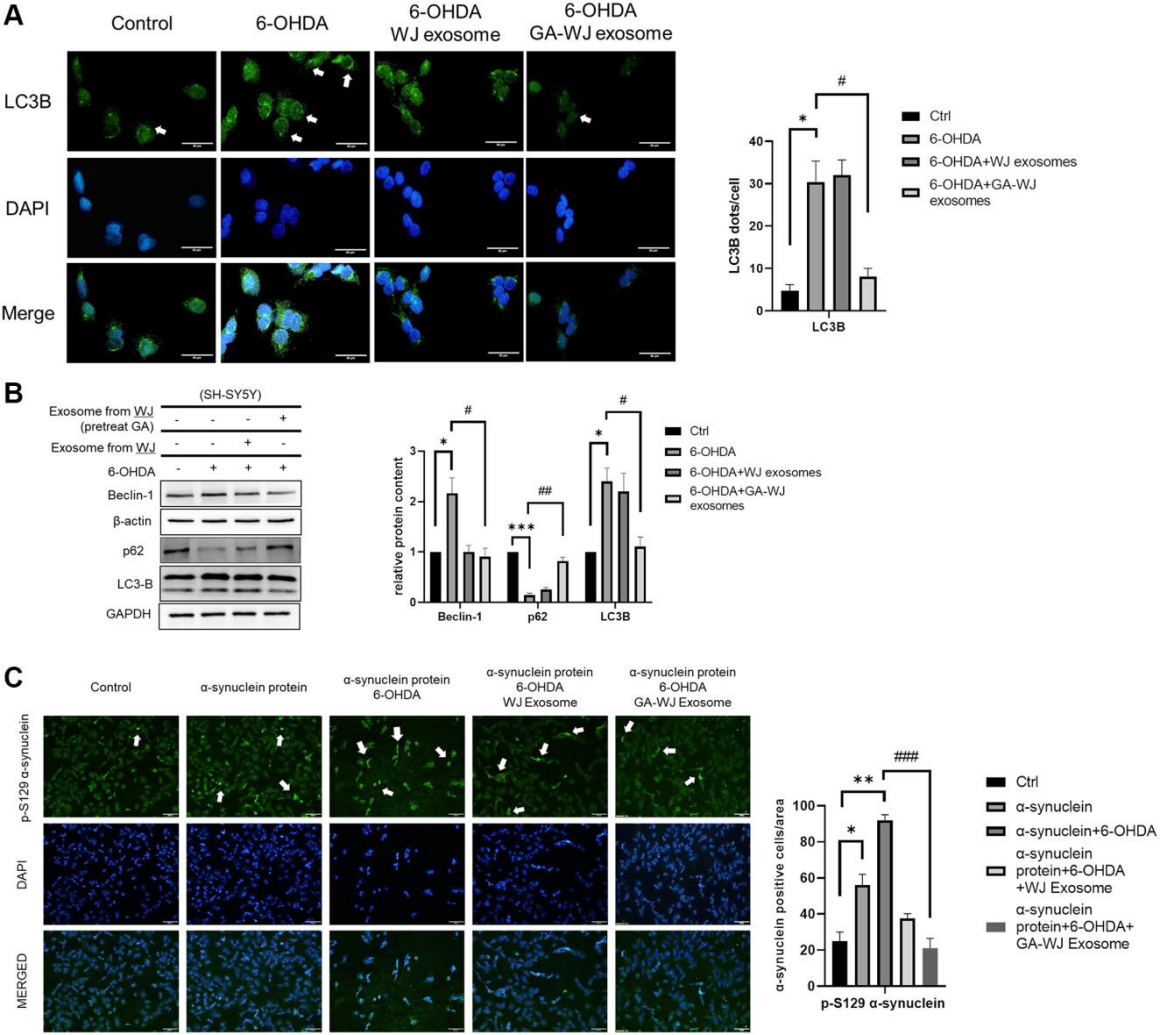
**GA pre-treated WJMSCs exosomes repair excessive autophagy in 6-OHDA stimulated model.** (**A**) The intensity and puncta of LC3-B were measured by immunofluorescence and DAPI staining. The white arrows indicate the dots showing increased puncta. (**B**) Immunoblot assays were performed to examine the Beclin-1, p62, and LC3-B proteins in the 6-OHDA damage model. (**C**) Immunofluorescence staining with alpha-synuclein p-129 anti-body after alpha-synuclein recombinant protein treatment in 6-OHDA damage model in SH-SY5Y cells. The white arrows indicate the expression of alpha-synuclein p-129. ^*^P < 0.05, ^**^P < 0.01, ^***^P < 0.001 vs. control and ^#^*P <* 0.05, ^##^*P* < 0.01, ^###^*P* < 0.001 vs. 6-OHDA groups.

6-OHDA treatment in neuronal cells leads to abnormal autophagic flux and causes cell apoptosis [23]. Hence, we investigated the autophagic flux in the cell damage model. The LC3 dots were abundant under 6-OHDA treatment. However, the puncta in SH-SY5Y cells can be recovered similar to that of control after treatment with the GA pre-treated WJMSC derived exosomes (Figure 6A). Immunoblotting assay showed higher Beclin-1 and LC3B but reduced p62 protein expression under 6-OHDA stimulation. Treatment with the exosomes from GA pre-treated WJMSCs restored the autophagic dysregulation similar to that observed in the control group (without 6-OHDA) (Figure 6B). To further validate our findings, we treated the SH-SY5Y cells with alpha-synuclein (5 μM, active, Pre-Formed Fibrils, GeneTex) under 6-OHDA stimulation which led to increased alpha-synuclein aggregation in the 6-OHDA group. However, GA pre-treated WJMSCs-derived exosome treatment significantly reduced the alpha-synuclein aggregation, suggesting that it could promote alpha-synuclein clearance through improved autophagic flux (Figure 6C).

## DISCUSSION

**Figure 7:**
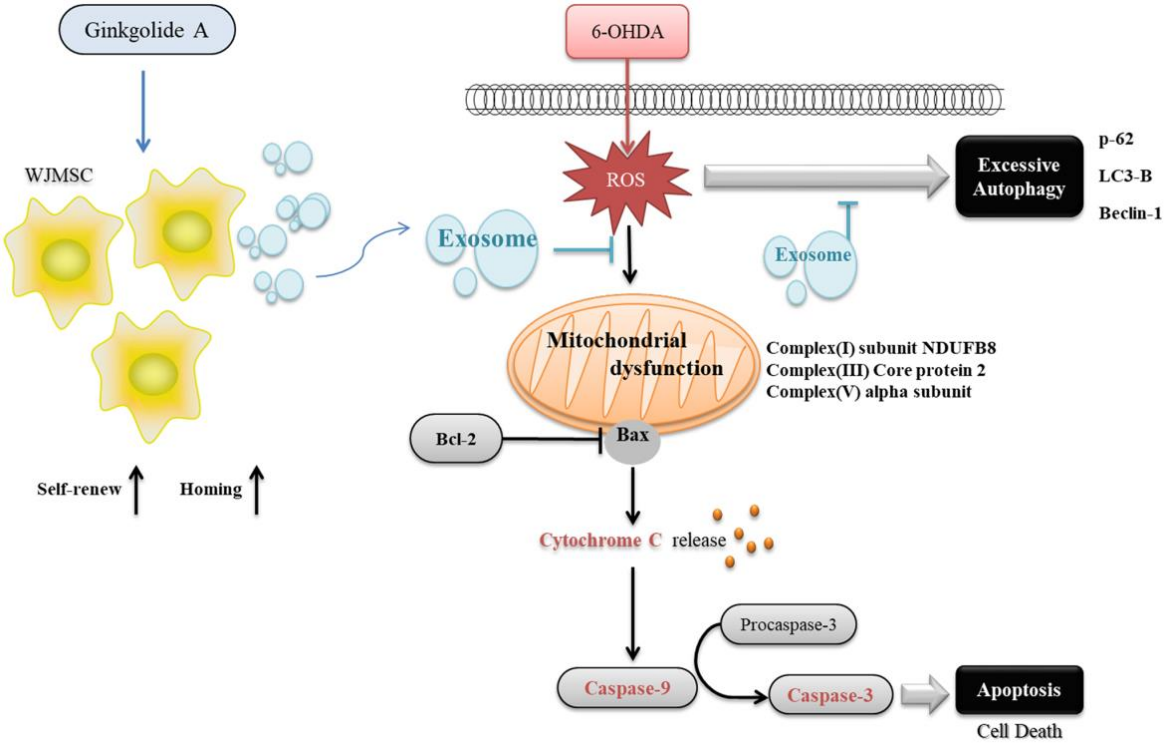
The graphical abstract of Ginkgolide A reinforcing multiple functions of WJMSC.

Most of the Ginkgolide derivatives from *Ginkgo biloba* are known for their properties such as anti-inflammatory, neuroprotective, and anti-cancer effects [24–26]. GA, GB, and GC could protect against cerebral ischemia/reperfusion damage in rats through Nrf2/CREB regulation [27]. GB promoted neuronal differentiation through the Wnt/β-catenin pathway in neural stem cells from mice [28]. It was hence applied in several platforms for enhancing cell differentiation and Parkinson's model [29–32]. Although various functions of GA have been determined, such as anti-atherosclerosis by preventing oxidative stress [33]; and anti-neurotoxic by preventing Tau hyperphosphorylation [17], direct evidence showing GA function in stem cells is lacking. Our study is the first to contribute to revealing that GA has potential benefits like GB in stem cells but in promoting stemness rather than cell differentiation. Therefore, GA can provide a new strategy to maintain stem cell properties for clinical application. In this study, we demonstrated a novel finding to use the natural compound GA that promoted the proliferation, self-renewal, and homing ability of WJMSCs, and further enhanced the exosomes secreted from WJMSCs to repair 6-OHDA-induced dopaminergic neuronal cell death. GA pre-treated WJMSC-derived exosomes could not only inhibit 6-OHDA induced excessive autophagy by regulating autophagy-related protein thus recover mitochondrial complex I/III/V but balancing the autophagy functions in alpha-synuclein clearance and protecting the cell from apoptosis (Figure 7).

Stem cell therapy is popular due to its self-renewal ability and differentiation into various cell lineages. It brings hope for the treatment of diseases which cannot be cured by drug therapy. However, there are some disadvantages that need to be overcome such as the allogenic graft-dependent rejection. The paracrine effect of the cytokines or the secretome and immunomodulation is more important than the MSCs themselves. Exosomes derived from different types of stem cells have been utilized in the treatment of numerous disease models [34]. Using compounds/components with a specific function in regenerative medicine gave rise to its unique worth, which could improve its outcome. The study showed that exosomes from Fisetin-treated keratinocytes could promote hair growth in an animal model [35]. Adiponectin stimulated exosome production by hMSCs to protect against heart failure in mice [36]. Here, we found that GA can enhance the function of exosomes from WJMSCs to mitigate cell death in a Parkinson’s disease cell model, providing a better outcome than those isolated from untreated WJMSCs.

The Parkinson’s disease *in vitro* models that include MPP+ and 6-OHDA treatment in neuroblastoma cells are well established with known mechanisms. Exosomes isolated from WJMSCs have been shown to repair cell damage caused by 6-OHDA-induced autophagic dysfunction [22]. Several studies also highlighted that 6-OHDA led to excessive autophagy that resulted in the release of cytochrome *C* from the mitochondria [23]. In our study, we found the exosome derived from GA pre-treated WJMSCs can return excessive autophagy to the normal state, thereby restoring OXPHOS (Figures 5C, 6A and 6B). 6-OHDA treatment in SH-SY5Y induced LC3/Beclin 1 but decreased p62 expression which confirmed autophagy activation in our model. However, exosome derived from GA pre-treated WJMSCs reversed the autophagy-related protein expression implying that the exosomes could inhibit autophagy thus protect neuronal survival. Interestingly, GA pre-treated WJMSCs derived exosomes brought about a significant clearance of the alpha-synuclein protein in 6-OHDA treatment (Figure 6C). These data reveal that the protective effects of exosomes might be attributed to their efficiency in stabilizing autophagy even under 6-OHDA stimulation. Therefore, analyzing the contents of the exosomes is key to ascertain the potential components (such as proteins, mRNAs, or non-coding RNAs from the donor MSCs) and mechanisms that mediate their beneficial effects. The average size of GA pre-treated WJMSCs exosome was larger than that of the control MSCs (Figure 4A). It might hint to the presence of more “cargo,” which is promoted by GA treatment in the WJMSCs to secrete or aid in exosome biogenesis. The specific and potential targets in GA pre-treated WJMSCs exosome with a high repair capacity will be examined in future experiments.

## CONCLUSION

We found a potential compound, GA, that can not only promote WJMSCs stemness and homing ability but also enhance exosome function to repair Parkinson’s disease *in vitro*. The beneficial effects were mediated through GA’s restoration of autophagy function to clear the alpha-synuclein protein and repair mitochondrial dysfunction (Figure 7). This study aids in providing evidence for implementing stem cell-derived exosomal therapy in the PD treatment regime.
